# Lipid Nanoparticles for mRNA Delivery to Enhance Cancer Immunotherapy

**DOI:** 10.3390/molecules27175607

**Published:** 2022-08-31

**Authors:** Hong-Li Wang, Zhi-Gang Wang, Shu-Lin Liu

**Affiliations:** 1 Engineering Research Center of Nano-Geomaterials of Ministry of Education, Faculty of Materials Science and Chemistry, China University of Geosciences, Wuhan 430074, China; 2 School of Medicine, College of Chemistry, Nankai University, Tianjin 300071, China

**Keywords:** lipid nanoparticles, mRNA delivery, cancer immunotherapy

## Abstract

Messenger RNA (mRNA) is being developed by researchers as a novel drug for the treatment or prevention of many diseases. However, to enable mRNA to fully exploit its effects in vivo, researchers need to develop safer and more effective mRNA delivery systems that improve mRNA stability and enhance the ability of cells to take up and release mRNA. To date, lipid nanoparticles are promising nanodrug carriers for tumor therapy, which can significantly improve the immunotherapeutic effects of conventional drugs by modulating mRNA delivery, and have attracted widespread interest in the biomedical field. This review focuses on the delivery of mRNA by lipid nanoparticles for cancer treatment. We summarize some common tumor immunotherapy and mRNA delivery strategies, describe the clinical advantages of lipid nanoparticles for mRNA delivery, and provide an outlook on the current challenges and future developments of this technology.

## 1. Introduction

Cancer is currently a crucial cause of death among patients and an expanding number of people around the world are dying from cancer [1,2]. It causes a very severe threat to human health, and most cancer patients are at an advanced stage when diagnosed, often producing a poor prognosis. Therefore, research on cancer treatment has become a focus of attention. For cancer treatment, traditional treatments include surgery, radiotherapy, and chemotherapy, but cancer treatments are not effective and can have significant side effects. Immunotherapy, which has emerged in recent years, is considered to be a promising approach to overcome cancer. It artificially activates the immune system by regulating and controlling the working mechanisms of the immune system, rather than targeting the tumor itself, in order to clear malignant tumor cells. Available therapies are cancer vaccines [3,4,5], adoptive cell therapy (ACT) [6,7,8,9], immune checkpoint blockade (ICB) [10,11,12,13,14], and cytokines [15]. Cancer immunotherapy is well established and current immune agents include vaccines, T cell activators, dendritic cell (DC) stimulators, and immune checkpoint inhibitors [16], whose therapeutic principle is mainly T cell-regulated antitumor immunity, but the effector function of T cells is not autonomous, and effective immune responses may be negatively regulated by tumor regulatory T cells (Tregs) influence [17,18]. In addition, infiltrating lymphocytes have difficulty achieving cytotoxic effects against core regions of solid tumors in the immunosuppressed tumor microenvironment (TME), and immunotherapy for some tumor patients faces significant clinical challenges, including resistance to agents, low immunogenicity, severe immune-related adverse events (iRAEs), non-targeted systemic toxic effects, and high treatment costs [19].

Messenger RNA (mRNA) plays an important role in tumor immunotherapy by effectively delivering cytokines, costimulatory receptors, or therapeutic antibodies and is well suited for cancer vaccines and neoantigen vaccination [20,21,22]. However, there are still some challenges with mRNA delivery systems in terms of targeted delivery and endosomal escape, and therefore, safer and highly efficient mRNA delivery strategies have a great impact on the immunotherapy efficacy in the evolving cancer immunotherapy. Many materials have been developed that can be used for mRNA delivery, and notably, lipid nanoparticles are excellent nanodrug carriers for mRNA delivery during cancer immunotherapy, which is a hot topic in tumor therapy at present [23]. A number of lipid nanoparticle–mRNA formulations for the prevention or treatment of various diseases have been investigated and are undergoing clinical studies. Different materials have different delivery efficiencies for mRNA and exhibit different accumulation effects and cancer cell uptake rates at tumor sites [24,25]. Therefore, lipid nanoparticles have been widely studied by many researchers as ideal immune carriers for regulating mRNA delivery and have entered clinical applications in tumor immunotherapy.

In this paper, we briefly introduce some typical lipid nanoparticles for mRNA delivery, describe some of the difficulties encountered in this research process, and finally, we present examples of excellent mRNA delivery systems for enhancing tumor immune effects in clinical studies, and provide an outlook on the future prospects of lipid nanoparticles and tumor immunotherapy.

## 2. The Emerging of Cancer Immunotherapy

**Figure 1:**
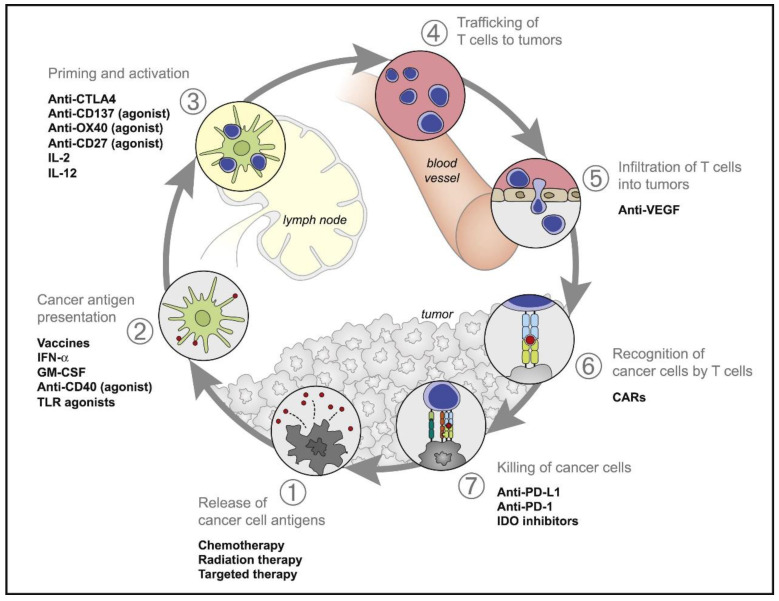
Schematic diagram of the tumor immune cycle. Stimulating and inhibiting factors in the immune cycle, and replacement therapy affecting the cycle [30].

**Figure 2:**
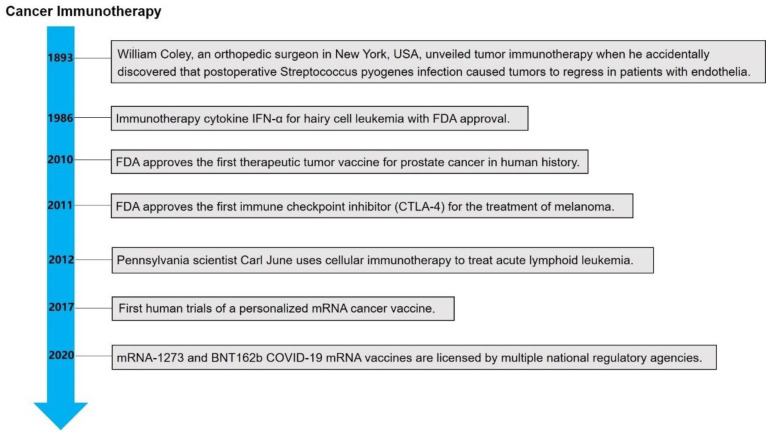
Timeline of some key milestones for cancer immunotherapy development.

Cancer immunotherapy is a method that uses immunological principles to enhance immunity. It is able to inject immune cells and effector molecules into the host, which can stimulate or enhance the antitumor immune effect and inhibit the growth of tumors. In 1986, the U.S. Food and Drug Administration (FDA) agreed to the use of the immunotherapeutic cytokine interferon-α (IFN-α) for the therapy of hairy cell leukemia, and several clinical trials ranging from cytokines to interleukin-2 (IL-2) have demonstrated some efficacy and a high level of toxicity [26,27]. Many studies have shown that immunotherapy is effective for certain cancers, demonstrating promising applications for a variety of immunotherapies to improve treatment outcomes, and the FDA has approved immunotherapy for the therapy of non-small cell lung cancer, kidney cancer, bladder cancer, liver cancer, head and neck cancer, cervical cancer, and melanoma [28]. In 2013, *Science* ranked tumor immunotherapy in the top 10 scientific breakthroughs, and immunotherapy is expected to be the next generation of tumor treatment after surgery, chemotherapy, and radiotherapy [29]. In previous studies, cancer immunotherapy targeted immunosurveillance mechanisms and did not directly target tumor cells. These steps include tumor-associated antigen release, antigen-presenting cell presentation, T cell initiation and activation, T cell migration and infiltration, T cell discovery and clearance on the tumor, and the action of certain costimulatory factors (Figure 1) [30,31,32,33]. Based on these mechanisms, cancer immunotherapies can be divided into the following categories: cytokines, cancer vaccines, ICBs, and ACTs. In addition, innovations in delivery systems have facilitated the study of personalized mRNA vaccines, providing a favorable rationale for mRNA vaccines as a promising cancer immunotherapy (Figure 2).

Cytokines are a specific class of molecules secreted by immune cells that act by binding with high affinity to target cell surface receptors, thereby regulating cellular functions [34,35,36]. Cytokines include lymphokines produced by lymphocytes and mononuclear factors produced by mononuclear macrophages. Currently known cytokines include interleukin (IL), interferon (IFN), tumor necrosis factor (TFN), etc. Research on the corresponding receptors, biological functions and clinical applications of these cytokines has become an important area of clinical immunology. During the immune response, cytokines play an important role in cell–cell interactions, cell growth, and differentiation, but can also lead to pathological responses under abnormal conditions. It has been shown that cytokine release syndrome (CRS) follows 5 days of BTN162b2 (an mRNA vaccine of coronavirus disease 2019) vaccination in a long-term colorectal cancer patient receiving anti-PD-1 therapy [37]. CRS is a systemic inflammatory response characterized by elevated inflammatory markers, thrombocytopenia, and excessive release of cytokines (i.e., INF-γ, IL-6, IL-10, and IL-2R are elevated) [38,39,40]. Oncolytic virus (OV) therapy is a novel form of cancer treatment developed in recent years that enables the selective removal of cancer cells using natural or engineered viruses [41], with viral genomic modifications to enhance antitumor activity and attenuate pathogenicity [42]. OV is frequently modified to express unique cytokines that enhance immune cell aggregation and activation, or to cause tumor cells to produce costimulatory molecules that enhance the costimulatory effect of T cells by facilitating the expression of T cell activation signals [43]. OV is a less toxic option compared to other cancer immunotherapy strategies, but still has some limitations, for example, OV-regulated antitumor responses are likely to have an impact in immunocompromised patients [41]. In addition, IFN-α is effective in hematological malignant diseases such as hairy cell leukemia, but is less effective in solid tumors. TNF is currently being attempted for local application in rectal cancer due to serious systemic side effects and efficacy checks, but the exact efficacy needs to be further evaluated. Therefore, the development of appropriate and effective new therapies for clinical use is a major issue for future cancer treatment.

Cancer vaccines, which use tumor cell-associated antigens to activate the immune system, can recognize proteins on specific cancer cells, thus killing cancer cells without harming normal cells, and showing good preventive and therapeutic effects to stop cancer cell growth. Cancer vaccines not only generate novel antigen-specific T cell responses, but also amplify existing responses, thereby focusing the host’s immune response on tumor cells [44,45]. Unlike chemotherapy and radiotherapy, which directly kill tumor cells and rapidly divide normal cells in the body, cancer vaccines usually do not have serious side effects and can induce an immune response in “cold” tumors that are not immunogenic themselves, thus potentially transforming them into “hot” tumors. With the continued development of preventive vaccines such as hepatitis B virus (HBV) and human papillomavirus (HPV), HPV vaccines have shown some potential for cancer prevention, dramatically reducing HPV prevalence and precancerous lesions and saving millions of lives [46,47,48,49,50,51,52,53,54,55,56,57,58,59,60,61]. The goals of therapeutic cancer vaccines are to induce tumor clearance, establish durable antitumor memory, and reduce adverse reactions [62]. Early therapeutic vaccination strategies focused on self-antigens that are aberrantly expressed or overexpressed in tumors, called tumor-associated antigens (TAAs)—but TAA-specific T cells are affected by central and/or peripheral tolerance—lack tumor specificity and have poor immunogenicity [63]. However, therapeutic vaccines have been slow to develop and face many challenges [64,65,66,67,68,69,70,71,72,73,74,75,76]. To break this tolerance, cancer vaccines must load a large number of tumor antigens onto DCs. Among them, the specific recognition of tumor-associated carbohydrate antigens (TACAs) has promising applications in tumor immunotherapy. Cancer vaccines achieve favorable therapeutic effects by inducing antigen-specific CD8^+^ cytolytic T cells (CTLs) and antigen-specific CD4^+^ T cells, which are mainly determined by the capture and presentation of antigens by DCs [77,78,79,80]. It has been shown that N-hydroxyneuraminic acid (Neu5Gc), a dietary carbohydrate, generates new antigens upon sustained accumulation on human tumor cells. In a mouse model, passive immunotherapy with anti-Neu5Gc antibodies suppressed the increase in size of Neu5Gc-positive tumors. Using engineered α-blood knockout porcine erythrocytes expressing Neu5Gc-TACA bionanoparticles in their natural environment, researchers developed a therapeutic mechanism for an active cancer vaccine against Neu5Gc-positive tumors and, after optimizing adjuvant and immunization procedures, applied these bionanoparticles to inoculated “human-like” Neu5Gc-deficient mice and found that this evoked a robust and durable anti-Neu5Gc immune effect and suppressed the growth of tumor volume [81]. The results of many clinical trials have shown that cancer vaccines are an effective and precise antitumor immunotherapy with relatively little harm to the body, but tumor-induced immunosuppression and immune resistance remain a major challenge, and safer and more effective cancer vaccines need to be developed to treat cancer by overcoming tumor resistance and improve clinical outcomes [82,83,84].

ICB is a method for triggering antitumor immune responses that blocks cancer cell-activated immunosuppression and has demonstrated significant efficacy in the treatment of many types of tumors [85,86,87,88,89,90]. Human cancers carry multiple somatic mutated genes and epigenetic variant genes that produce substances often recognized by the immune system as antigens. PD-1 is an expressed inhibitory receptor produced by antigen-stimulated T cells that regulates and controls T cell proliferation, cytokine release, and cytotoxicity. PD-L1 receptors act on tumor cells and bone marrow-derived suppressor cells [91]. As an adaptive checkpoint, PD-L1 recognition of the PD-1 receptor significantly reduces T cell activation, inhibits antitumor effects, and fails to kill tumor cells normally. Inhibition of the PD-L1/PD-1 pathway restores the antitumor immune response, thereby enhancing the host immune system’s aggressiveness against tumor cells [92]. CD47 is an innate immune checkpoint that interacts with receptor signaling regulatory protein α (SIRPα), thereby inhibiting phagocytosis by macrophages, and inhibition of the CD47/SIRPα signaling pathway may also limit tumor growth [92,93]. It has been shown that inhibition of proprotein convertase subtilisin/kexin type 9 (PCSK9), an important protein in the regulation of cholesterol metabolism, can enhance the effects of immune checkpoint therapy. Deletion of the PCSK9 gene in tumor cells enhances the effectiveness of immunotherapy against PD-1 and prevents the growth in tumor size. In addition, inhibition of PCSK9 by gene deletion or other methods promotes the action of major histocompatibility protein class I (MHC I) proteins on cancer cells and enhances tumor infiltration of T cells [94,95]. However, single ICB has disadvantages such as less effective response and potentially higher side effects, insufficient reversal of TIM, and complete clearance of tumor cells [96,97,98,99]. Due to the limited efficacy of ICB against hepatocellular carcinoma (HCC) and other cancers, researchers have developed a targeted mRNA nanoparticle platform designed to induce the expression of p53 (tumor suppressor gene) in HCC models. Combining p53 mRNA with anti-PD-1 therapy effectively inhibits the growth of tumor volume [100].

ACT is an important method for immune cell therapy of infiltrative tumors, using the antitumor characteristics of lymphocytes to remove primary and metastatic tumor cells [101,102,103,104,105,106,107,108,109,110,111,112,113,114,115,116,117]. Autologous (patient’s own) or allogeneic (donor’s) tumor-infiltrating lymphocytes (TILs) are activated in vitro, expanded to a certain number, and reinfused into the patient [118,119,120,121]. In recent years, the use of artificial T cells constructed from chimeric antigen receptors (CARs) and T cell receptors (TCRs) for various aspects of cancer therapy has progressed considerably. It was found that combining the DC growth factor FMS-like tyrosine kinase 3 ligand (Flt3L) secreted by T cells with the immune agonist poly (I:C) and anti-4-1BB, Flt3L-secreting T cells were found to increase the number of DCs within the tumor and significantly enhance T cell activity. Importantly, in tumor models, combined treatment with T cell receptor and chimeric antigen receptor T cells significantly inhibited tumor growth and induced the spread of antigenic epitopes beyond the already metastatic T cells [122]. It has been shown that retroviral gene transfer of IL-12 into T cells has shown serious side effects. To overcome this toxicity, transient genetic engineering with mRNA encoding IL-12 and CD137 ligands inhibited the growth of tumor volume and improved the therapeutic efficacy of ACT [123]. In addition, it has been demonstrated that CARs and TCRs move transiently by electroporation of in vitro transcribed mRNAs optimized for gene expression [124,125]. In vitro gene transfer of synthetic mRNAs with electroporation devices is clinically feasible [126]. ACT is a more complex immunotherapeutic approach than others, and the “off-target” toxic effects of antigens on normal tissues remain a problem, so more intensive research is needed to improve this situation [127].

**Figure 3:**
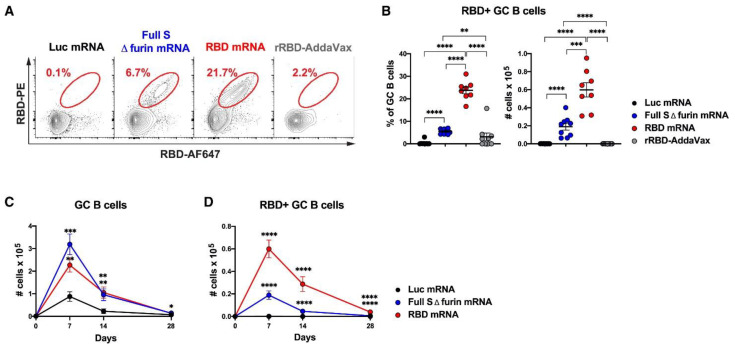
Antigenic response after intravenous administration of SARS-CoV-2 mRNA vaccine. (**A**) Flow cytometry counts of RBD-specific GC B cells. (**B**) Frequency and absolute number of cells after 7 days of immunization. (**C**) Kinetics of the absolute number of cells. (**D**) Curves of absolute cell numbers versus time [135]. * *p* ≤ 0.05, ** *p* ≤ 0.01, *** *p* ≤ 0.001, **** *p* ≤ 0.0001.

mRNA-based vaccines are being investigated as a means of encoding antigenic proteins and providing adjuvant functionality. The high potency of mRNA and the unprecedented speed of its development and manufacture have made mRNA vaccines promising therapeutic approaches that have shown great clinical potential and saved millions of lives [128]. In contrast to immune checkpoint blockade therapies targeting PD-1, PD-L1, or CTLA-4, mRNA vaccines are able to attack “non-self” cancer cells by inducing T and B cells. During the treatment of various hematological malignancies, the activity of malignant B cells is depleted and may impair the humoral (B cell) response induced by the mRNA vaccine [129]. For example, studies in mice with multiple sclerosis have shown that immunization with mRNAs encoding self-antigens and delivered as non-inflammatory liposomal carriers can suppress autoimmunity by activating antigen-specific regulatory T cells [130]. Successful application of mRNA vaccines against SARS-CoV-2 demonstrated good tolerability, but their instability and inefficient delivery limit the antitumor effect (Figure 3) [131,132,133,134,135]. In addition, an mRNA-based multitumor epitope approach is able to stimulate effective antitumor immunity against tumor antigens in melanoma patients. However, the application of mRNA vaccines is inhibited by the inability to bind effectively to immune adjuvants. Lipid nanoparticles enable agents to exhibit specific accumulation effects and uptake by cancer cells at tumor sites, and are excellent nanodrug carriers for cancer immunotherapy processes. Therefore, mRNA vaccines with lipid nanoparticles as carriers can achieve long-lasting anticancer effects, and the development of therapeutic mRNA vaccines will drive oncology research forward.

## 3. Development of Lipids for mRNA Delivery

In the past few years, mRNA has made considerable progress in cancer immunotherapy, but the instability and high immunogenicity of mRNA in vivo have hindered the translation into clinical practice [136,137,138]. To address this challenge, mRNA modification and delivery techniques have been studied in greater depth and it has been found that lipids play a significant role in mRNA delivery, both in conferring protection from mRNA degradation and in improving cellular transfection [138,139]. Lipids contain three structural regions, the polar headgroup, the hydrophobic tail region, and the linker between the two structural regions. Many studies have shown that cationic lipids, ionizable lipids, and other lipids are increasingly being used for mRNA delivery with favorable results [140,141,142].

### 3.1. Cationic Lipids

Cationic lipids generally consist of positively charged polar groups and hydrophobic tails, and are capable of self-assembling into higher order aggregates in aqueous solution. Cationic lipids are an ideal vector for good targeting, low side effects, good stability, and high transfection efficiency. Cationic lipids can interact with negatively charged lipids in biological membranes to form a membrane-disrupting non-bilayer structure that can allow nucleic acid polymers to enter the cell, thereby facilitating mRNA delivery [143,144]. 1,2-dioleoyl-3-trimethylammonium-propane (DOTAP) and 1,2-di-O-octadecenyl-3-trimethylammonium-propane (DOTMA) are commonly used as cationic lipids, which have poor stability, membrane fusion, and transfection efficiency when forming liposomes alone. DOTAP and DOTMA have been used alone or in combination with other materials to transfer mRNA to various cell types [145,146,147], and the fusion and transfection efficiency were improved with the addition of the auxiliary lipids such as 1,2-dioleoyl-sn-glycero-3-phosphoethanolamine (DOPE) and 1,2-dioleoyl-sn-glycero-3-phosphocholine (DOPC). The mRNA and cationic lipids can form stable complexes, and the amino groups of cationic lipids usually interact with the phosphate groups in nucleic acid molecules, which can be encapsulated in lipid nanoparticles for tumor immunotherapy, showing good immunogenicity [148,149,150]. For example, dimethyldioctadecylammonium bromide (DDAB) is a cationic lipid that stimulates the immune response and becomes stable when bound to mRNA, thus acting as an adjuvant for mRNA vaccines, and is capable of improving the effectiveness of immunotherapy [151,152]. In addition, DOTAP-based cationic nanoemulsions can be used to deliver antigenic mRNA against multiple types of infections, and DOTAP–polymer hybrid nanoparticles can transfer mRNA for tumor immunotherapy [153,154,155].

Although cationic lipid delivery systems appear promising, cationic lipids can be neutralized by anionic serum proteins during mRNA delivery, reducing mRNA delivery efficiency as well as therapeutic efficacy [156].

### 3.2. Ionizable Lipids

**Figure 4:**
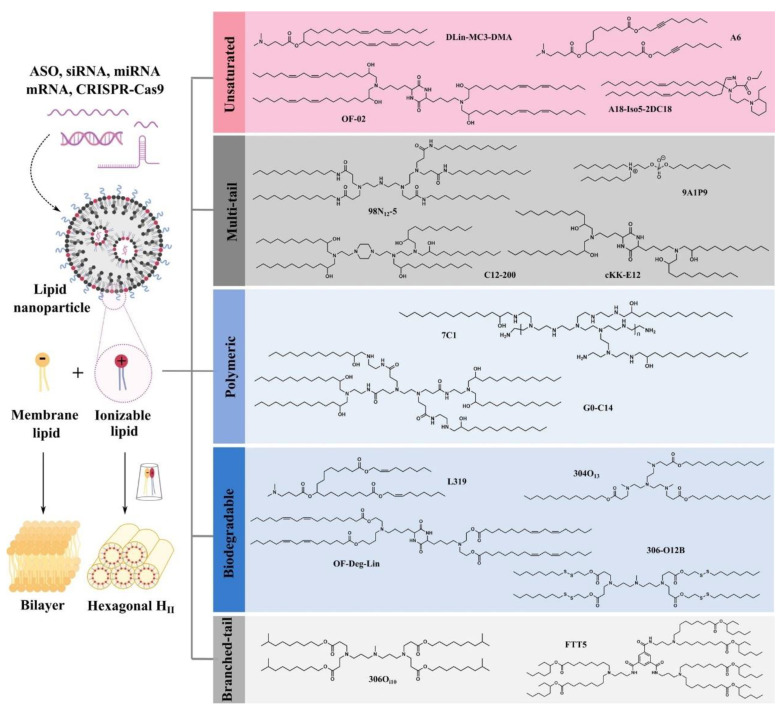
Mechanism of endosome destruction by ionized lipids. Based on their structural characteristics, ionized lipids can be classified into five types: unsaturated, multitailed, polymerized, biodegradable, and branching tails [165].

Ionizable lipids are amphiphilic structures with hydrophilic head groups that promote self-assembly of hydrocarbon chains, as well as linkers between head groups and hydrocarbon chains. Ionizable lipids play an important role in protecting RNA and facilitating cytoplasmic translocation and are capable of protonation at low pH, making these lipids positively charged and capable of improving stability and reducing systemic toxicity, but are usually uncharged at physiological pH [157]. pH-sensitive ionizable lipids facilitate mRNA delivery in vivo and often exhibit better biocompatibility [158]. In endosomes with low pH, ionizable lipids can be protonated, which improves the fusion of these lipids with the endosomal membrane and promotes endosomal escape as well as mRNA migration to the cytoplasm. Therefore, the effect of ionizable lipids on mRNA delivery efficiency depends on the pH at which they are protonated and the ability of ionizable lipids to form non-bilayer structures. The introduction of ionizable lipids enhances the role of mRNA in vivo, and the addition of ionizable lipids not only maintains mRNA delivery efficacy but also achieves rapid metabolism, improves the tolerance of lipid nanoparticles, and enhances the effectiveness of tumor immunotherapy (Figure 4) [159,160,161,162,163,164,165]. For example, researchers have developed an ionizable lipid material to facilitate mRNA delivery in vivo and to provide an effective immune-activated mRNA delivery vehicle to stimulate a strong immune response and inhibit the growth of tumor volume [166]. Recently, a variety of ionizable lipids have been created for different applications, greatly facilitating the further development of mRNA delivery. It has been shown that unsaturated ionizable lipids can enhance mRNA delivery, with linoleic acid-derived ionizable lipids (OF-02) showing better liver mRNA delivery and more significant protein expression compared to their counterparts. However, the unsaturated bonds contained in ionizable lipids do not always correspond to efficient delivery of mRNA in vivo, suggesting that design and screening are a very important step [165]. To minimize side effects, the introduction of biodegradability in ionizable lipids is a common strategy, and lipids are often degraded to non-toxic metabolites at the end of intracellular delivery, which is particularly important for RNA therapies that require repeated administration. Compared to non-degradable analogues, ester-containing ionizable lipids tend to exhibit lower potency due to low delivery efficiency, therefore, a balance between activity and degradability is needed to maximize benefits.

### 3.3. Other Types of Lipids

In addition to common lipid types, mRNA formulations often include other lipids such as phospholipids, cholesterol, or polyethylene glycol lipids (PEG-lipids), which can improve the properties of mRNA preparations such as biodistribution, stability, and delivery efficiency [167]. 1,2-distearoyl-sn-glycero-3-phosphocholine (DSPC) is a phosphatidylcholine with a unique geometry that allows for better stability of lipid nanoparticles and enhances therapeutic efficacy by promoting the fusion of lipid nanoparticles with cellular and endosomal membranes and enhancing cellular uptake and endosomal release. Notably, DSPC is also involved in the production of mRNA-1273 and BNT162b2 COVID-19 vaccines [168,169]. Cholesterol, known as a regulator of membrane fluidity, fills the gaps between lipids within membranes and can improve the stability of lipid nanoparticles by controlling membrane integrity and rigidity [170], and derivatives of its molecular geometric configuration can more profoundly affect mRNA delivery efficiency and biodistribution. For example, since the length of the hydrophobic tail, the flexibility of the sterol ring, and the polarity of the hydroxyl group of cholesterol analogues affect the delivery efficiency, cholesterol analogues containing C-24 alkyl phytosterols can improve the delivery efficiency of mRNA drugs in vivo [171]. PEG-lipids consist of a PEG molecule conjugated to alkyl chains that anchor themselves in the bilayer of lipid nanoparticles. PEG-lipids are important in mRNA delivery, binding ligands to particles for targeted delivery, reducing the permeation of serum proteins and clearance by reticuloendothelial cells, resulting in more potent delivery of mRNA, more drug accumulation at tumor sites, and better immunotherapeutic efficacy [172,173].

In conclusion, mRNA has promising applications as a genetic medicine that induces transient protein expression, promotes a wide range of biological processes, and reduces the risk of genomic integration [174]. However, the existing mRNA delivery systems do not meet the demand and greatly hinder the clinical progress of mRNA therapies. Therefore, a more in-depth study of lipids and the application of lipid nanoparticles in the mRNA delivery process is needed.

## 4. Lipid Nanoparticles for mRNA Delivery in Cancer Immunotherapy

The unique advantages of nanomaterials have led to their widespread application in cancer therapy [175,176]. Many gold nanoparticles, inorganic nanoparticles, and lipid nanoparticles have been investigated to deliver therapeutic drugs to cancer cells through passive targeting mechanisms or active targeting mechanisms. For example, phospholipid nanoparticles (PL1) were found to be effective in delivering costimulatory receptor mRNA (CD137 or OX40) to tumor-infiltrating T cells, and the use of PL1-OX40 mRNA and anti-OX40 antibody was shown to have more significant antitumor activity than anti-OX40 antibody alone in a variety of tumor models [3]. Therefore, lipid nanoparticles are increasingly used in tumor immunotherapy, with significantly improved antitumor effects and greatly reduced systemic side effects [177,178]. Several clinical studies are using lipid nanoparticles for in vivo delivery of mRNA therapeutics, and fortunately, many lipid nanoparticles have successfully entered the clinic for mRNA delivery. For example, the coronavirus disease 2019 (COVID-19) vaccine uses lipid nanoparticles to deliver antigenic mRNA. Subsequently, we will present several common examples of lipid nanoparticles that enhance tumor immunotherapy by modulating mRNA delivery [179,180].

### 4.1. Liposomes

Liposomes are bilayers formed by spherical phospholipids and cholesterol as the main components [181,182,183,184,185,186], with the advantages of high encapsulation rate, good targeting, and low toxicity, which have promising applications in industrial production. Hydrophilic small-molecule drugs can be enclosed in an internal aqueous core, while hydrophobic agents are enclosed in a lipid bilayer [187], and the encapsulation of therapeutic drugs in distinct liposome chambers allows for safe and targeted drug delivery and protects the encapsulated cargo from being cleared by the immune system. Liposomes are one of the approaches to enhance cancer immunotherapy by modulating mRNA delivery, which can deliver hydrophilic and lipophilic therapeutic drugs while maintaining efficacy.

**Figure 5:**
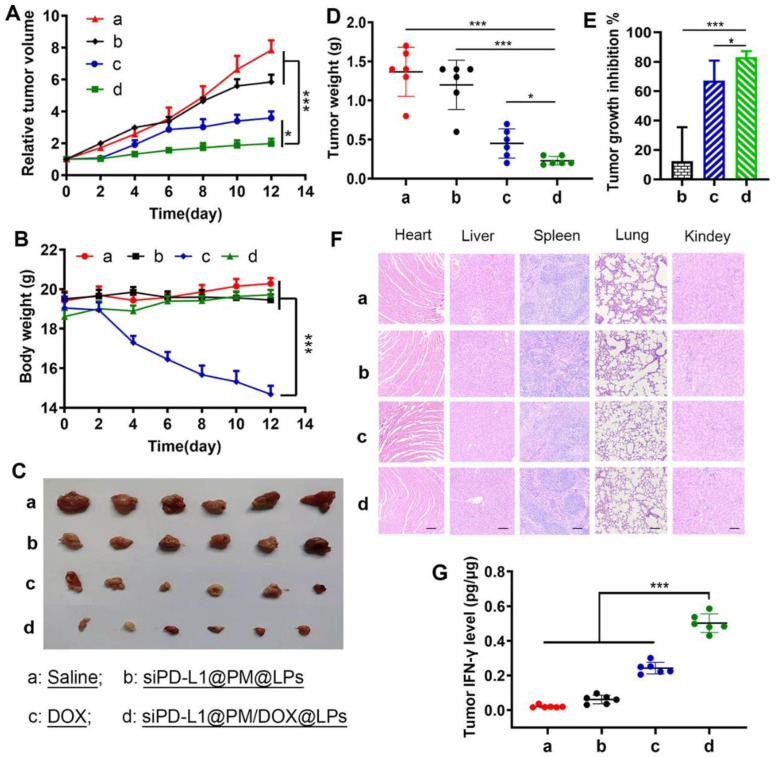
Antitumor effects in vivo ((**a**) saline; (**b**) siPD-L1@PM@LPs; (**c**) free DOX; and (**d**) siPD-L1@PM/DOX@LPs). (**A**) Tumor growth curve. (**B**) Weight change during the experiment. (**C**) Pictures of tumor tissues isolated after the completion of the experiment. (**D**) Tumor weight change curve with time. (**E**) Tumor growth inhibition rate under different conditions. (**F**) H&E stain images of major organs (scale bar: 50 μm). (**G**) Levels of cytokine IFN-γ in tumor tissues [199]. * *p* ≤ 0.05, *** *p* ≤ 0.001.

The combination of liposomal drugs with immunotherapeutic agents is a promising immunotherapeutic approach in which the immunotherapeutic agent is enclosed inside the liposome, improving the application of immunotherapeutic drugs as they are released. Some liposomal agents such as Doxil^®^, LipoTaxen^®^, Onivyde^®^, and Taxol^®^ have achieved promising therapeutic results in clinical practice. As a commonly used mRNA delivery system, encapsulating mRNA in liposomes protects mRNA from degradation by nucleases, aids cellular uptake, and promotes endosomal escape [188]. Some studies have shown that PD-L1 inhibitors are a common immunotherapeutic agent targeting cancer cells [189,190,191,192,193,194,195,196,197,198], and the multifunctional liposomal nanocarriers siPD-L1@PM/DOX/LPs have excellent stability in serum and can effectively deliver siRNA into MCF-7 cells to reduce PD-L1 expression and enhance immunotherapeutic effects (Figure 5) [199]. Thus, immunotherapy continues to develop and increasingly more immunotherapies will be explored in depth. For example, immunoliposomes are a novel immunotherapeutic approach for mRNA delivery [200], which generally couples antibodies to the surface of liposomes and works together with chemotherapeutic drugs enclosed in liposomes to enhance the immune function of the body, accelerate the immune response, and improve the chance of liposome coupling to the target site. Long-circulating liposomes can increase flexibility and hydrophilicity due to PEG modification, reduce the interaction between liposomal lipid membranes and plasma proteins through phagocytosis by the monocyte–macrophage system, prolong circulation time, and facilitate targeting of tissues or organs other than the liver and spleen, while binding antibodies or ligands at the end of PEG can maintain the recognition of the target.

In recent years, novel liposomes for mRNA delivery have emerged, and liposomes as drug carriers are an early class of novel targeted agents for clinical application, but few have finally entered clinical trials, and further improvements in lipid type, binding bonds, and binding rates of chemotherapeutic drugs and antibodies are needed before they can be used in clinical translation of cancer therapy.

### 4.2. Nanodiscs

**Figure 6:**
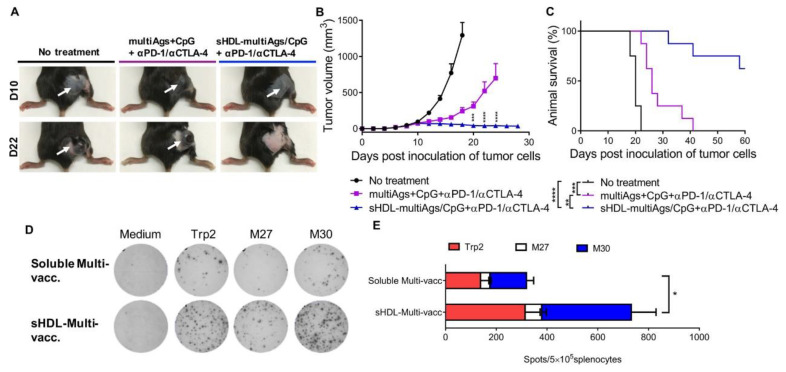
Therapeutic effects of the combination of vaccine nanodiscs and immune checkpoint blockers. (**A**) Images of tumor-bearing mice at the beginning of treatment (day 10) and during treatment (day 22). (**B**) Mean tumor growth over time. (**C**) Animal survival over time. (**D**) Images of ELISPOT wells. (**E**) The number of tumor antigen-specific IFN-γ+ spots [204]. * *p* ≤ 0.05, ** *p* ≤ 0.01, *** *p* ≤ 0.001, **** *p* ≤ 0.0001.

The efficacy of many cancer agents at the clinical stage is unsatisfactory, and various nanosystems have been investigated with great success in order to improve the antitumor effect [201,202]. Nanodiscs are a synthetic model membrane system consisting of phospholipid bilayers surrounded by proteins or polymers, which can be used as novel nanomaterials for immunotherapy. Their structure is similar to that of discoidal high-density lipoproteins and better mimics the natural environment than liposomes and micelles. The process of intravenous drug delivery often requires the addition of solubilizers to improve utilization, but it can easily lead to problems such as high injection doses and toxic side effects. In nanodiscs, because they mimic the phospholipid bilayer of biological membrane, the hydrophilic head of phospholipid molecules is exposed on the outside, the long lipophilic chains are located inside the nanodisc structure, the loaded drugs are wrapped in the middle of the internal long lipophilic chains, and many experiments have proved that nanodisc materials have better loading ability for lipophilic drugs [203]. For example, researchers developed personalized vaccine nanodiscs of HDL for delivery of immunostimulants and antigens, and when the nanodiscs were used in combination with PD-1 and cytotoxic T-lymphocyte-associated protein 4 (CTLA-4) to treat mice with advanced B16F10 melanoma tumors, the combined immunotherapy exerted a powerful antitumor effect, eradicating established tumors in approximately 60% of the animals (Figure 6) [204]. Nanodiscs as drug carriers have made great progress in applied research due to their advantages of controlled drug release, targeting function, and high drug loading rate. Nanodiscs customized with patient-specific tumor neoepitopes are a promising platform and a new approach for tumor immunotherapy, but they are currently limited to relevant tests in animal models and still need to be further explored before they can be expected to better exploit the advantages of drug carriers in the clinical setting.

### 4.3. Lipid–Polymer Hybrid Nanoparticles

**Figure 7:**
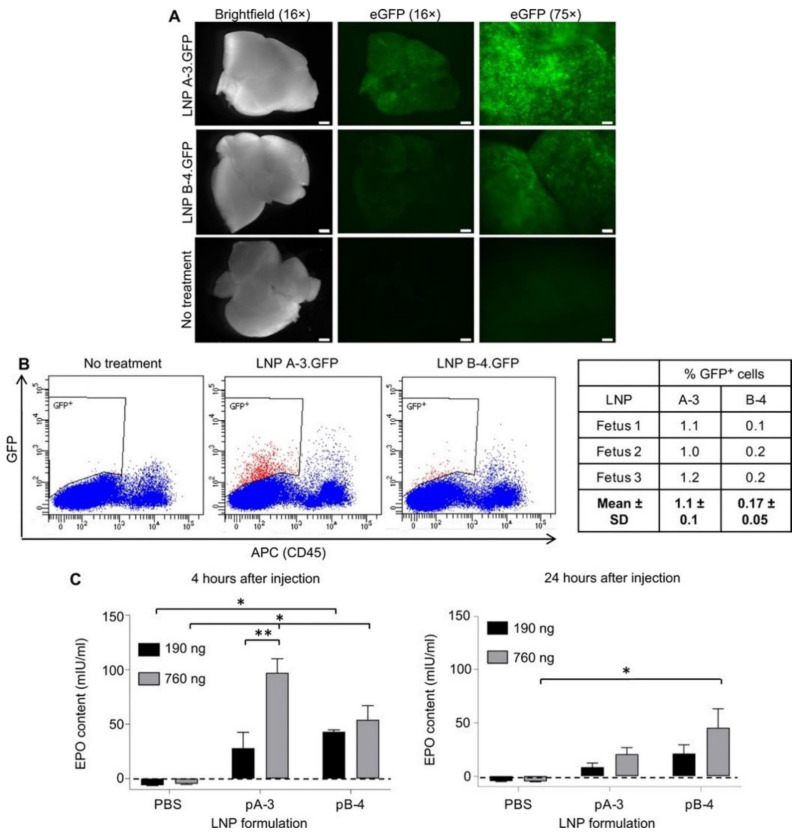
GFP mRNA and EPO mRNA were delivered in utero with LNPs. (**A**) GFP expression in fetal liver at 24 h post-injection. (**B**) Analysis of single cell suspensions from fetal liver by flow cytometry, recording the percentage of CD45^-^ and GFP^+^ cells. (**C**) Levels of EPO in fetal liver after injection [207]. * *p* ≤ 0.05, ** *p* ≤ 0.01.

Lipid–polymer hybrid nanoparticles are a class of scalable and biodegradable nanocarriers that show significant potential for mRNA delivery [205]. Attractive for immunotherapy due to their structural versatility, inorganic nanoparticles with lipid shells have been investigated to effectively encapsulate drugs [206]. LPN is a mature technology platform for safe and effective delivery of RNA drugs. Compared with other nucleic acid drug delivery systems, LPN has great advantages, such as high nucleic acid encapsulation rate and effective cell transfection, high tissue penetration, low cytotoxicity and immunogenicity. For example, researchers have used materials such as ionizable lipid libraries, phospholipids, cholesterol, and lipid-anchored PEG to create a lipid–polymer hybrid nanoparticle that can effectively deliver mRNA to mouse fetuses [207,208]. After encapsulation of mRNA by lipid–polymer hybrid nanoparticles and intravenous injection into the fetus, it was demonstrated that lipid–polymer hybrid nanoparticles enabled functional delivery of mRNA to the liver, lungs, and intestine. Notably, lipid–polymer hybrid nanoparticles were also used to deliver erythropoietin (EPO) mRNA to demonstrate its therapeutic potential. Delivery of EPO mRNA to mouse fetal hepatocytes increased the EPO protein content in the fetus and greatly enhanced the therapeutic effect (Figure 7) [207].

Lipid–polymer hybrid nanoparticles retain the properties of lipid nanoparticles while providing more structural options that offer advantages in immunotherapy. Therefore, LNPs are a key technology for mRNA vaccines to effectively protect mRNA and transport it into cells to play an important role, but their complex structures and manufacturing processes can hinder the clinical application of these heterogeneous nanoparticles [209], and further research is needed to overcome these hindrances to achieve better therapeutic results.

### 4.4. Micelles

In recent decades, mRNA has developed into a very effective therapeutic method [210,211], but the stability of mRNA remains a limiting factor for its efficiency [212]. Micelles are molecularly ordered aggregates that start to form massively after the surfactant concentration in aqueous solutions reaches a certain value. The hydrophobic groups aggregate to form the inner micelle core, avoiding contact with polar water molecules. The hydrophilic groups form the outer layer of the micelle, which can interact with water molecules and protect the internal groups. The compounds forming micelles are generally amphiphilic molecules that are soluble in polar solvents such as water. Biocompatible polylactic acid (PLA)-based micelles offer safe and degradable advantages, and some investigators designed a micelle-based mRNA delivery platform that combines PLA-based micelles and cationic dense peptides to provide a new option for clinical applications [213]. Relying on the coupling of RALA peptides (histidine-/arginine-rich amphiphilic peptides) on micelles [214,215,216], mRNA was further captured by electrostatic interactions. Thus, micelles were found to adequately protect mRNA from degradation by serum nucleases, decrease the toxic effects of cationic peptides, and facilitate transfection of DCs with significantly improved therapeutic efficacy.

## 5. Conclusions and Future Directions

In the last few decades, lipid nanoparticles have enabled a dramatic improvement in mRNA delivery for enhancing tumor immunotherapeutic efficacy and have attracted wide interest in the biomedical field. In order to achieve the desired application of lipid nanoparticles, intensive preclinical and clinical research on the properties of lipid nanoparticles is needed when studying their immune formulation. (1) Lipid nanoparticles have good targeting properties, which can improve the effectiveness of drugs and reduce the occurrence of toxic side effects. Specific ligands at the focal site can be coupled to lipid nanoparticles, so that these nanoparticles interact specifically with tumor cells and deliver drugs to specific sites in a timely manner. (2) The composition of lipid nanoparticles is similar to cell membranes and has good cellular affinity and histocompatibility, and can be adsorbed around target cells for a long time, allowing the drug to fully penetrate into the target cells and enter the cells through fusion. (3) Encapsulating drugs in lipid nanoparticles can attenuate the excretion and metabolism of drugs in the kidneys and increase the drug half-life, thus enhancing the effect of drugs. (4) Lipid nanoparticles are often used in new combination therapies, which can improve the synergistic effect of combination therapies in tumor treatment by controlling the release of small molecule drugs in the body at the appropriate rate and concentration through diffusion and permeation. It can improve the synergistic effect of combination therapy in tumor treatment, control the release of small molecule drugs in the body at appropriate rates and concentrations through diffusion and permeation, effectively improve the bioavailability of insoluble drugs, reduce the degree of drug damage to normal tissues, and improve the effectiveness of treatment.

Therefore, the application of lipid nanoparticles in mRNA delivery for tumor immunotherapy has received much attention and achieved many exciting immunotherapeutic results, providing much valuable information for future tumor immunotherapy. Given the rapid development of lipid nanoparticles in recent years and the remarkable application potential shown in several clinical trial phases, the next generation of lipid nanoparticles will be further developed for tumor immunotherapy, thus improving healthcare and bringing new hope for the treatment of various diseases.

## Data Availability

No new data were created or analyzed in this study. Data sharing is not applicable to this article.
